# Superimposed Training Combined Approach for a Reduced Phase of Spectrum Sensing in Cognitive Radio

**DOI:** 10.3390/s19112425

**Published:** 2019-05-28

**Authors:** Lizeth Lopez-Lopez, Marco Cardenas-Juarez, Enrique Stevens-Navarro, Ulises Pineda-Rico, Armando Arce, Aldo G. Orozco-Lugo

**Affiliations:** 1 Facultad de Ciencias, Universidad Autónoma de San Luis Potosí (UASLP), San Luis Potosí 78290, Mexico; lizeth.lopez.lpz@gmail.com (L.L.-L.); estevens@galia.fc.uaslp.mx (E.S.-N.); u.pinedarico@gmail.com (U.P.-R.); 2 Cátedras CONACYT, Universidad Autónoma de San Luis Potosí (UASLP), San Luis Potosí 78290, Mexico; armando.arce@uaslp.mx; 3 Centro de Investigación y de Estudios Avanzados del Instituto Politécnico Nacional (CINVESTAV-IPN), Ciudad de México 07360, Mexico; aorozco@cinvestav.mx

**Keywords:** spectrum sensing, superimposed training, cognitive radio

## Abstract

This paper presents an approach to exploit the superimposed training (ST)-based primary users’ (PUs) transmissions in the context of spectrum sensing for cognitive radio. In the low signal-to-noise ratio (SNR), the proposed scheme splits the spectrum sensing phase into two sample processing periods, allowing a secondary user (SU) to carry out a training sequence synchronization (with a small probability of error) before the implementation of a robust spectrum sensing algorithm that enhances the detection, based on the deterministic signal components embedded in the ST PU’s signals along with the unknown data signal. The overall sensing performance is improved using a reasonable number of samples to achieve a high probability of detection, resulting in a reduced spectrum sensing duration. Furthermore, a low computational complexity version of the proposed ST combined approach for a reduced phase (SCAR-Phase) of spectrum sensing is presented, which attains the same detection performance with a smaller number of real operations in the low SNR. In the practical consideration of imperfect training sequence synchronizations, the results show the advantages of exploiting the ST sequence to perform spectrum sensing, thus quantifying the significant improvement in detection performance and the maximum SU’s achievable throughput.

## 1. Introduction

Cognitive radio (CR) is envisioned as one of the technologies that will alleviate the demand for more radio frequency (RF) spectrum required by future wireless communications systems and networks. In CR networks, the shift is towards the implementation of opportunistic spectrum access mechanisms, in which a frequency band can be accessed not only by its spectrum license holder, i.e., the primary user (PU) but also by secondary users (SUs) in a non-intrusive way [1,2]. In other words, SUs are allowed to access a licensed frequency band only when the PUs’ signals are idle either in time or geographic location [3]. Hence, the RF spectrum availability can be improved given that the underutilized frequencies can be exploited by SUs, creating the need for different approaches [4]. One of these is spectrum sensing, which must be performed by SUs to know the available frequency bands by detecting the PUs’ transmissions.

The most popular spectrum sensing algorithm is the energy detector, which does not need to know any information about the PUs’ signals (i.e., blind spectrum sensing) and stands out for its computational simplicity and promptness. However, its detection performance rapidly deteriorates under noise power uncertainty [5] and in the low signal-to-noise ratio (SNR), where a high number of samples are required to attain a certain detection performance [6]. Alternatively, other blind spectrum sensing algorithms have been proposed to overcome the shortcoming of the energy detector, such as Maximum-to-Minimum Eigenvalue, Maximum Eigenvalue Detection, Covariance Absolute Value, and Covariance Frobenius Norm [7]. These algorithms are based on the statistical covariance or auto-correlation of the received signals, which increases the computational complexity since they require a high number of samples. Therefore, their performance drastically deteriorates if the number of received samples is small. In contrast, pilot (or training) symbols, commonly included in the PUs’ transmitted signals for synchronization and channel estimation purposes, can be used to improve the detection of PUs if such patterns are known by the SUs. Indeed, previous investigations have demonstrated the significant improvement in terms of detection performance achieved by algorithms that combine training and data sequences to perform spectrum sensing, especially in the low SNR region [8,9,10,11,12]. For example, in [8], an enhanced detector for real-valued PUs’ signals with embedded pilots (e.g., digital TV signals) is proposed for spectrum sensing over a lossless channel. This detector takes advantage of both the known pilot symbols and the energy of the received signal to improve the detection performance of SUs. In [9] a semi-blind spectrum sensing algorithm is designed by exploiting the knowledge of a training sequence that is time-multiplexed with binary phase shift keying (BPSK) modulated PU’s signals. Interestingly, the detector consists of the linear combination of the matched filter output, the energy, and pseudo-energy of the received signal, thus enhancing the detection performance of SUs while keeping a relatively low computational complexity. Alternatively to time-multiplexed training sequences, in [12] it is considered that low-power training information is superimposed (i.e., added) to the PU’s data prior transmission for its own convenience. Hence, a detector for superimposed training (ST)-based PUs’ signals buried in noise is designed. The results show that the detection performance improves significantly even with very low-power training sequences. However, a lossless channel is considered in the design of the detector, which is not the case in a practical wireless scenario.

Furthermore, in order to take advantage of the training sequence, a realistic model for spectrum sensing implies to consider other important aspects. Amongst these, two major concerns can be distinguished: (i) SUs need to know the training information about the PUs’ signals, and (ii) the synchronization of the SU with the PU system is required. Regarding the former, however, it is possible for the SUs to obtain information about the PUs’ signals if both users operate under a cooperation agreement in the CR context. For example, recent studies have considered PUs that opt to share part of their spectral resources with the SUs to get relaying services [13] or to improve the overall spectral efficiency, amongst other sorts of rewards [14]. Therefore, in such a scenario, it is also possible to share the PU’s signal information, such as the modulation type or pilot sequences. Regarding the latter, the training information of the PUs can be used by the SUs to synchronize their receivers with the PU if a certain amount of time is assigned for this purpose.

In this paper, a new approach that allows SUs to exploit the training sequence using a synchronized received sequence is proposed to improve the energy detector. After the sample collection of the spectrum sensing phase, the new method uses a first sample processing period to perform a synchronization procedure at the time the energy detection is carried out since it does not require the SU to be synchronized with the PU’s transmissions. Then, in a second sample processing period, the new combined approach utilizes the synchronized sequence to implement an enhanced training-based detection algorithm, as long as the energy detector decides the absence of the PU’s signal. Hence, the focus is on the spectrum sensing of superimposed training (ST)-based PU’s transmissions, given the fact that ST signals might benefit both PUs and SUs. On the one hand, PUs’ receivers can improve their own channel estimation task without reducing the data transmission bandwidth, which is of great importance for high data rate transmissions [15,16]. On the other hand, SUs can consider the known ST sequence in the design of spectrum sensing algorithms to improve their detection performance, which reduces the unwanted interference to PUs’ transmissions and increases the SUs’ throughput. Additionally, ST allows the implementation of robust synchronization algorithms at the SUs’ receivers, like those introduced in [17,18]. Therefore, a detector for ST PUs’ signals (called ST-Det) is designed and used in the second sample processing period of the proposed superimposed training combined approach for a reduced phase (SCAR-Phase) of spectrum sensing to implement an enhanced detection, considering the synchronized training and the data sequences. Furthermore, in order to reduce the computational complexity, a simplified version of the SCAR-Phase of spectrum sensing is presented. The proposed schemes are useful in the very low SNR region, where synchronization with the PU can be achieved to improve the spectrum sensing algorithms by exploiting the ST sequence. In order to show how the energy detector can be improved by means of the ST sequence in the very low SNR region, the proposed approaches are compared with this spectrum sensing algorithm. The results show that both, SCAR-Phase and simplified SCAR-Phase, requires a significantly reduced number of samples to achieve a target probability of detection in the low SNR, in contrast to the energy detector. Moreover, the SU’s throughput gain is quantified for the proposed methods.

The rest of the paper is organized as follows: In Section 2 the spectrum sensing scenario under consideration is described for ST PU’s transmissions and the detection problem is formulated. In Section 3 the proposed SCAR-Phase of spectrum sensing is detailed along with the design of a detector for ST-based PUs’ signals under a flat fading channel. The performance metrics of the SCAR-Phase of spectrum sensing are presented in Section 4. In Section 5, a simplified version of the SCAR-Phase spectrum sensing is described. Moreover, the results are shown in Section 6. Finally, Section 7 concludes the work.

## 2. Superimposed Training System Model and Detection Problem

In the PU’s network it is assumed that a low power training sequence is superimposed (i.e., added) to the data signal at the transmitter, which allows the PUs’ receivers to improve the synchronization and channel estimation tasks. In the SU’s network the ST information can be exploited to improve the spectrum sensing function. Moreover, the ST technique is suitable to overcome the synchronization requirement that comes with the use of the deterministic signal components of coherent processing, which has been scarcely investigated in the context of CR. For this, it is assumed that the PU shares its training information with the SUs’ receivers according to the statutes of a previous cooperation agreement. Indeed, this can be done for profit or to benefit both users in the cognitive radio scenario, as it has been recently contemplated in [14] for different cooperation schemes. The ST PUs’ transmitted signal and the spectrum sensing problem formulation at SU’s receivers are explained in the following section.

### 2.1. Superimposed Training PU’s Transmitter

**Figure 1:**
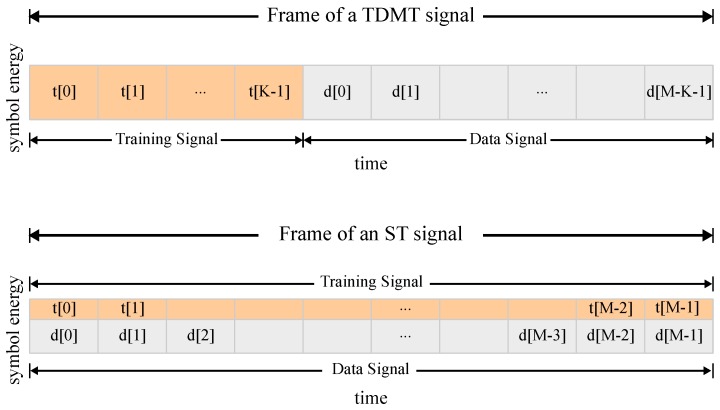
Comparison of frame structures for the time domain multiplexed training (TDMT) scheme (**top**) and the superimposed training (ST) scheme (**bottom**).

In practical wireless communications systems, training symbols known by the receiver are used to perform channel estimation and synchronization for reliable transmissions. Traditionally, separate time slots are used to transmit training symbols independently of the data symbols, which is known in the literature as the time domain multiplexed training (TDMT) scheme. This, certainly, requires the allocation of a fraction of the total bandwidth for the training sequence, as visualized in Figure 1 (top). As a result, the data rate of the PU to transmit signals in a specific frequency band is reduced. Alternatively to TDMT, the ST technique consists in adding a low-power periodic training sequence to the PU’s data sequence before it is transmitted, as visualized in Figure 1 (bottom), increasing its data transmission bandwidth at the cost of requiring some additional processing [16]. In addition, the PU’s receivers also achieve better synchronization and channel estimation by means of the ST sequence; as a consequence, its bit error rate is decreased.

The PU’s transmitted sequence is given by [15]

(1) $$\begin{array}{c} s[n]=t[n]+d[n], \end{array}$$

where t[n] denotes the ST sequence and d[n] denotes the data (or information) sequence. The ST sequence is a non-random periodic sequence with period *P* and average power given by $\sigma_{t}^{2}=\left(1/P\right)\sum_{n=0}^{P-1}{\left|t\left[n\right]\right|}^{2}$. Note that *N* represents the sample index. Moreover, the data sequence is assumed to be zero mean with average power denoted by $\sigma_{d}^{2}$. Generally, an ST system is characterized by the training-to-information ratio (TIR) value, which is defined as $\alpha =\sigma_{t}^{2}/\sigma_{d}^{2}$. Small values of TIR are usually specified for the PUs since the ST sequence acts as input noise on the data sequence. Therefore, it is assumed that the PU sends the ST-based signal through a frequency band selected from those available for its data transmissions. In the SU network, the receivers must identify the idle bands, thus formulating the detection problem explained in the next section.

### 2.2. Detection Problem for SUs in Cognitive Radio

In order to determine the available frequency bands for opportunistic transmissions, SUs rely on the spectrum sensing functionality. For the CR scenario analyzed here, it is considered that in a frequency band of the PU, each SU independently performs the spectrum sensing task over *N* received samples, with N/P being an integer. Hence, the vector $x={\left[x\left[0\right],\;x\left[1\right],\;\cdots \;,x\left[N-1\right]\right]}^{T}$ denotes the sequence periodically collected by the SU’s receiver, with each sample of the set expressed as:

(2) $$\begin{array}{cc} x\left[n\right]=\theta e^{j\phi }s\left[n-\tau \right]+w\left[n\right], & n=0,\;1,\;\ldots ,\;N-1, \end{array}$$

where θ and ϕ represent the unknown amplitude and phase shift of a flat fading channel, respectively. Furthermore, s[n−τ] denotes the independent and identically distributed (i.i.d.) ST PU’s signal samples given by Equation (1) with power $\sigma_{s}^{2}$, where τ is used to express the lack of time synchronization between the PU’s transmitter and the SU’s receiver. Then, it is assumed that the transmitted data sequence d[n] is unknown to the SU and the samples are modeled as i.i.d. circularly symmetric complex Gaussian random variables with variance $\sigma_{d}^{2}$, i.e., $d\left[n\right]∼\mathrm{CN}\left(0,\;\sigma_{d}^{2}\right)$ and the ST sequence t[n] is known to the SU. Similarly, it is considered that the noise samples w[n] are i.i.d. circularly symmetric complex Gaussian random variables with zero mean and variance $\sigma^{2}$, i.e., $w\left[n\right]∼\mathrm{CN}\left(0,\;\sigma^{2}\right)$. Therefore, the detection problem consists in deciding between the following two hypotheses:

(i) The PU is not using the frequency band of interest (i.e., hypothesis $H_{0}:\;\theta =0$),
(ii) the PU is transmitting data (i.e., hypothesis $H_{1}:\;\theta >0$).

Nevertheless, deciding if the PU is active from the received sequence ${\left\{x\left[n\right]\right\}}_{n=0}^{N-1}$ can be very tough when random channel gains are considered. Especially in a very low SNR region (i.e., when θ→0), which results in collisions with the PUs (caused by missed detections) or in the loss of opportunities for SUs’ transmissions (caused by false alarms) due to the imperfect spectrum sensing. Additionally, exploiting the ST information for spectrum sensing requires the synchronization of the SUs in the CR network with the ST sequence transmitted by the PU, i.e., τ in Equation (2) must be estimated at each SU’s receiver. These aspects are considered in the next section, where a new superimposed training combined approach for a reduced phase (SCAR-Phase) of spectrum sensing is presented. This approach provides a feasible solution to the synchronization issue for spectrum sensing algorithms that take into account the training sequence to improve the detection performance in CR networks.

## 3. SCAR-Phase of Spectrum Sensing

Commonly, the energy detector has been widely studied to accomplish spectrum sensing in CR due to its low computational complexity and simplicity of implementation. However, its performance considerably degrades under noise power uncertainty. Besides, in the low SNR region, the required number of samples to achieve a target detection performance dramatically increases as the SNR decreases [19], which reduces the maximum SUs achievable throughput. In order to reduce the spectrum sensing phase without compromising the detection performance, the PU’s ST sequence can be used to improve sensing in the low SNR by combining the energy detector along with an enhanced ST-based detector. Additionally, the ST information can be used to synchronize the SU with the PU’s transmissions.

**Figure 2:**
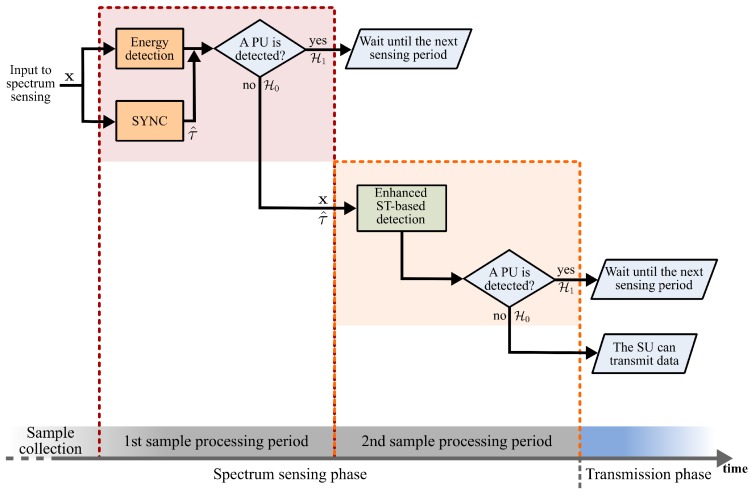
Block diagram of the proposed ST combined approach for a reduced phase (SCAR-Phase) of spectrum sensing.

The proposed SCAR-Phase of spectrum sensing block diagram is shown in Figure 2. The scheme considers that spectrum sensing is performed periodically followed by the SUs’ transmissions if the frequency band is idle. The SCAR-Phase of spectrum sensing consists of a sample collection period and two sample processing periods. Hence, after the SU collects the samples, in the first sample processing period a synchronization process (SYNC block in Figure 2) with the transmitted PU’s ST sequence is carried out at the same time that the energy of the received signal is calculated to make a first decision about the presence or absence of the PU’s signal. If a PU’s signal is detected (i.e., hypothesis $H_{1}$ is true), the SU waits until the next spectrum sensing phase. Otherwise (i.e., hypothesis $H_{0}$ is true), the second sample processing period is performed. During this period, the already synchronized ST sequence can be exploited through an enhanced ST-based detection that uses the set of collected samples. If an enhanced ST-based detector decides that the PU’s signal is present, the SU waits until the next spectrum sensing phase, or else the SU can transmit data. The first and second sample processing periods are explained in more detail next.

### 3.1. First Sample Processing Period

#### 3.1.1. Training Sequence Synchronization

The training sequence synchronization process represents a challenge for the SU, who must synchronize with the PU’s ST sequence and then decide the presence or absence of the PU in a very short time. The synchronization problem of ST-based transmissions has been previously studied in some investigations [17,18], which have proposed robust algorithms that can be used in the proposed approach to complete this task.

In this paper, a synchronization algorithm is implemented based on a particular case of the method proposed in [18], which exploits the characteristics of the cyclic mean of the received signal and has lower computational complexity than other methods. Hence, for the SU’s received signal model in (2), the time offset τ is estimated by choosing the integer $\hat{\tau }=\tau$ modulo-*P* (with $0\leq \hat{\tau }\leq P-1$) that satisfies the objective function given by

(3) $$J\left(\hat{\tau }\right):=\left|\right|{\left(C_{\hat{\tau }}^{-1}y\right)}_{{\left[P-1\right]}_{r}}\left|\right|=0,$$

where the matrix $C_{\hat{\tau }}$ is given by $C_{\hat{\tau }}=circ\left(t\left[-\hat{\tau }\right],t\left[-\hat{\tau }-1\right],\ldots ,t\left[-\hat{\tau }-P+1\right]\right)$, with circ(·) producing a circulant matrix. Moreover, the subscript ${\left[P-1\right]}_{r}$ indicates the last P−1 rows of a matrix and $y={\left[y\left[0\right],y\left[1\right],\ldots ,y\left[P-1\right]\right]}^{T}$ (with the superscript ${}^{T}$ denoting transpose) represents the period-P cyclic mean of Equation (2) given by

(4) $$y\left[j\right]:=E\left[x\left[iP+j\right]\right]=\theta e^{j\phi }t\left[j-\tau \right],$$

where E[·] denotes the expected value and j=0,1,…,P−1. This last sequence is expressed in matrix form as follows:

(5) $$y=C_{\tau }^{[1]c}\theta e^{j\phi },$$

where the matrix $C_{\tau }$ is given by $C_{\tau }=circ\left(t\left[-\tau \right],t\left[-\tau -1\right],\ldots ,t\left[-\tau -P+1\right]\right)$. Moreover, the superscript [1]c indicates the first column of the matrix $C_{\tau }$. Since τ is unknown, y must be estimated from the received signal at the SU. The proposed estimate $\hat{y}\left[j\right]$ (with j=0,1,…,P−1) is:

(6) $$\hat{y}\left[j\right]=\frac{1}{\left(N/P\right)}\sum_{i=0}^{(N/P)-1}x\left[iP+j\right],$$

which is used in (3) instead of (5). Given that (3) is only satisfied under ideal conditions (i.e., when θ and ϕ are perfectly known in a noiseless channel), $\hat{\tau }$ is therefore obtained by performing a linear search over $0\leq \hat{\tau }\leq P-1$ and then looking for the argument that minimizes $J\left(\hat{\tau }\right):=\left|\right|{\left(C_{\hat{\tau }}^{-1}\hat{y}\right)}_{{\left[P-1\right]}_{r}}\left|\right|$. That is,

(7) $$\underset{0\leq \hat{\tau }\leq P-1}{arg min}\;\;J\left(\hat{\tau }\right):=\left|\right|{\left(C_{\hat{\tau }}^{-1}\hat{y}\right)}_{{\left[P-1\right]}_{r}}\left|\right|.$$

**Figure 3:**
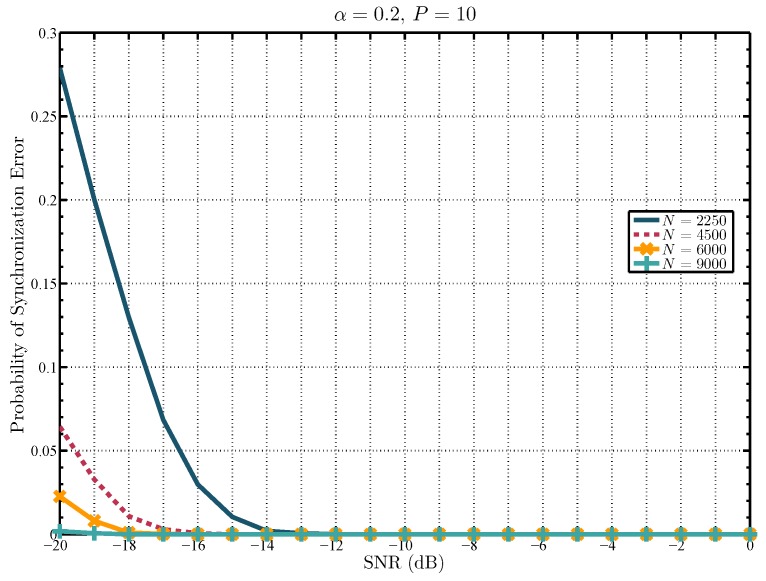
Probability of synchronization error against signal-to-noise ratio (SNR).

Note that, since y is estimated using the received signal x, the correct estimation of τ depends on the number of received samples *N*. Hence, the greater the number of received samples, the better the estimate of τ. The performance of the ST-based synchronization method is presented in Figure 3, where the probability of synchronization error is show against different values of SNR. The probability of synchronization error is found using 5000 Monte Carlo trials and a different number of samples. The received signal is modeled as in (2) with the value 0≤τ≤P−1 (τ∈N) varying in each Monte Carlo trial. Moreover, the training sequence is obtained as follows [15]:

(8) $$\begin{array}{ccc} t\left[n\right]=\sigma_{t}e^{j\frac{\pi }{P}\left[n\left(n+u\right)\right]} & u=1,P\;\mathrm{odd};\;u=2,P\;\mathrm{even}; & n=0,1,\ldots ,P-1, \end{array}$$

which has an unity peak-to-average power ratio, with period P=10 and $\sigma_{t}^{2}$ chosen such that $\sigma_{t}^{2}+\sigma_{d}^{2}=1$ and the TIR α=0.2. It can be noticed that for a determined value of SNR, the probability of synchronization error is lower when a greater number of received samples are utilized. It is important to note that in the SNR region of interest, up to −20 dB, the probability of synchronization error approaches zero with a reasonable number of samples *N*. For example, with N=6000, the probability of synchronization error is $\approx 2\times 10^{-2}$, and with N=9000 this probability reduces to $\approx 1.8\times 10^{-3}$. Therefore, Figure 3 shows that it is feasible for the SU to synchronize with the PU’s ST transmitted sequence using a relatively low number of samples.

#### 3.1.2. Energy Detection

At the same time the synchronization is performed, a first decision about the presence or absence of the PU’s signal can be made by means of an energy detector, since it does not require any prior information about the PU’s signal neither require synchronization. This allows the proposed approach to work according to the SNR level. That is, in the high SNR the operation of the energy detector might satisfy the minimum requirements of the PUs and SUs with a reasonable number of samples. Whereas in the low SNR, where the energy detector might exhibit a low detection performance and therefore require a very large number of samples, an enhanced ST-based detection can be implemented to improve the overall performance using a reduced number of samples once the synchronization has been realized.

The energy detector test statistic decides $H_{1}$ if

(9) $$\Phi_{ed}\left(x\right)=\sum_{n=0}^{N-1}{\left|x\left[n\right]\right|}^{2}>\lambda_{ed},$$

where $\lambda_{ed}$ is the threshold used to compared the test statistic. The probability of a false alarm of the energy detector is defined as $P_{\mathrm{fa}}^{ed}≜\mathrm{Pr}\left\{\Phi_{ed}\left(x\right)>\lambda_{ed}|H_{0}\right\}$, where Pr{·} denotes the probability operator. Hence, under the received signal model in (2) and for large *N* it is given by [20]

(10) $$P_{\mathrm{fa}}^{ed}=Q\left(\frac{\lambda_{ed}-N\sigma^{2}}{\sqrt{N\sigma^{4}}}\right),$$

where Q(·) is the Q-function or complementary cumulative distribution function of a standard Gaussian distribution [21]. Similarly, the probability of detection is defined as $P_{d}^{ed}≜\mathrm{Pr}\left\{\Phi_{ed}\left(x\right)>\lambda_{ed}|H_{1}\right\}$, which is given by

(11) $$P_{d}^{ed}=Q\left(\frac{\lambda_{ed}-N\theta^{2}\left(\sigma_{d}^{2}+\sigma_{t}^{2}\right)-N\sigma^{2}}{\sqrt{2N\theta^{2}\sigma_{t}^{2}\left(\theta^{2}\sigma_{d}^{2}+\sigma^{2}\right)+N{\left(\theta^{2}\sigma_{d}^{2}+\sigma^{2}\right)}^{2}}}\right).$$

Then, for a specific value of false alarm $P_{\mathrm{fa}}^{ed}=\beta$, the threshold can be obtained from (10) as:

(12) $$\lambda_{ed}=\sqrt{N\sigma^{4}}Q^{-1}\left(\beta \right)+N\sigma^{2},$$

which after its substitution in (11), gives the attained probability of detection for the β value.

### 3.2. Second Sample Processing Period: Enhanced ST-Based Detection

The second processing period is enabled when the SU does not detect the PU presence. This might be caused for several reasons, including its operation in the low SNR, the hidden terminal problem or missed detections of the energy detector. For the second processing period, it is proposed to take advantage of the PU’s training information once the training sequence synchronization process has been completed. For this task, an enhanced ST-based detector (ST-Det) is designed.

From the received signal model in (2), note that each sample of the set is distributed according to $x\left[n\right]∼\mathrm{CN}(\theta e^{j\phi }t\left[n-\tau \right],\;\theta^{2}\sigma_{d}^{2}+\sigma^{2})$. Since the received samples are considered to be i.i.d., the joint multivariate probability density function (or simply PDF) of x is given by

(13) $$p\left(x;\theta ,\phi \right)=\frac{1}{\pi^{N}{\left(\theta^{2}\sigma_{d}^{2}+\sigma^{2}\right)}^{N}}\exp\left(-\sum_{n=0}^{N-1}\frac{|x\left[n\right]-\theta e^{j\phi }{t\left[n-\tau \right]|}^{2}}{\theta^{2}\sigma_{d}^{2}+\sigma^{2}}\right),$$

which is said to be parameterized by the amplitude θ and the phase ϕ of the unknown channel gain.

The goal of a detector is to decide either $H_{0}$ or $H_{1}$ based on an observed set of data x. This is a mapping of each data set value into a decision [21]. In this sense, the locally optimum detection (LOD) criterion maximizes the slope of the power function (i.e., the probability of detection) amongst the detectors with the same probability of false alarm. Given that LOD produces relatively simple structures that work well in the low SNR region [22], it is used here in the design of the ST-Det that takes into account the PU’s ST information to enhance the overall detection of the SCAR-Phase of spectrum sensing. Hence, according to LOD, the SU decides $H_{1}$ is true when [23]

(14) $$\Phi \left(x\right)=\frac{{\left.p^{\left(i\right)}\left(x;\theta ,\phi \right)\right|}_{\theta =0}}{p(x;0,\phi )}>\lambda_{st},$$

where $p^{\left(i\right)}\left(x;\theta ,\phi \right)$ is the *i*-th derivative of p(x;θ,ϕ) with respect to θ, p(x;0,ϕ) is the PDF of x evaluated at θ=0 and $\lambda_{st}$ is a threshold against which the test statistic Φ(x) is compared. It is worth noting that p(x;θ,ϕ) evaluated at θ=0 is equal to

(15) $$p\left(x;0\right)=\frac{1}{\left(\pi^{N}\sigma^{2N}\right)}\exp\left(-\sum_{n=0}^{N-1}\frac{{\left|x\left[n\right]\right|}^{2}}{\sigma^{2}}\right),$$

which does not depend on the value of ϕ. Next, the first derivative of p(x;θ,ϕ) with respect of θ is obtained, which after some algebraic manipulations and rearranging some terms is found to be

(16) $$p^{\left(1\right)}\left(x;\theta ,\phi \right)=p\left(x;\theta ,\phi \right)\left\{\Lambda (\theta ,\phi )+\Omega (\theta )\right\},$$

where Λ(θ,ϕ) is equal to

(17) $$\begin{array}{cc} \Lambda (\theta ,\phi )= & \sum_{n=0}^{N-1}\left\{\frac{2\theta^{3}\sigma_{d}^{2}{\left|t\left[n-\tau \right]\right|}^{2}-4\theta^{2}\sigma_{d}^{2}ℜ\left\{e^{j\phi }t\left[n-\tau \right]x^{*}\left[n\right]\right\}+2\theta \sigma_{d}^{2}{\left|x\left[n\right]\right|}^{2}}{{\left(\theta^{2}\sigma_{d}^{2}+\sigma^{2}\right)}^{2}}\right.\\ & \left.-\frac{{2\theta |t\left[n-\tau \right]|}^{2}-2ℜ\left\{e^{j\phi }t\left[n-\tau \right]x^{*}\left[n\right]\right\}}{\theta^{2}\sigma_{d}^{2}+\sigma^{2}}\right\}, \end{array}$$

and Ω(θ) is given by

(18) $$\Omega \left(\theta \right)=-\frac{2N\theta \sigma_{d}^{2}}{\theta^{2}\sigma_{d}^{2}+\sigma^{2}}.$$

Note that ℜ{·} and ${}^{*}$ denote the real part and conjugate of a complex variable, respectively. Then, after evaluating (16) at θ=0 and substituting the result in (14), the first derivative-based test statistic is $\Phi_{{\mathrm{ST}}_{1}}\left(x,\phi ,\tau \right)=\left(2/\sigma^{2}\right)\sum_{n=0}^{N-1}ℜ\left\{e^{j\phi }t\left[n-\tau \right]x^{*}\left[n\right]\right\}$. In order to compute $\Phi_{{\mathrm{ST}}_{1}}\left(x,\phi \right)$, the parameters τ and ϕ must be estimated. Firstly, the estimate of τ modulo-P (i.e., $\hat{\tau }$) is obtained using (7). Secondly, it can be shown by using a Taylor series expansion of the likelihood function around θ=0, that

(19) $$\hat{\phi }=∠\sum_{n=0}^{N-1}t^{*}\left[n-\hat{\tau }\right]x\left[n\right]$$

can be used as a nearly maximum likelihood estimate for ϕ. It can be noticed that $\Phi_{{\mathrm{ST}}_{1}}\left(x,\phi ,\tau \right)$ also depends on the knowledge of the noise power $\sigma^{2}$, which in the ideal case is assumed to be perfectly known. However, in realistic scenarios, random noise distribution is never known entirely. Hence, in the strict sense, noise is neither completely stationary, white nor Gaussian [24]. Therefore, the noise power must be estimated by using a proper calibration method, whose output could be stored in the SU’s devices. For example, it can occur during the manufacturing process by replacing the antenna at the input of the SUs’ receivers with a matched load and collecting a set of noise samples every certain given time during a specified period [25]. However, this might not be precise since this value is different for a myriad of application environments. In consequence, accurate estimation of the noise power is not achieved in practice, thus leading to the noise power uncertainty problem that limits the performance of noise power-based sensing techniques [5]. Alternatively, in a practical implementation, $\sigma^{2}$ could be estimated by taking advantage of the silent periods in which the PU’s signal is not present and then, performing measurements periodically to update the noise statistics. The effect of noise power uncertainty in the proposed scheme is a matter of further investigation.

Since further simulations have shown a poor performance of $\Phi_{{\mathrm{ST}}_{1}}\left(x,\phi ,\tau \right)$, the use of the second derivative of p(x,θ,ϕ) with respect to θ in (14) is analyzed next to obtain the ST-Det for the second sample processing period. The second derivative of p(x;θ) is computed by deriving (16) with respect to θ, which after some algebra can be written as:

(20) $$p^{\left(2\right)}\left(x;\theta ,\phi \right)=p\left(x;\theta ,\phi \right)\left\{\Lambda^{\left(1\right)}\left(\theta ,\phi \right)+\Omega^{\left(1\right)}\left(\theta \right)+{\left[\Lambda (\theta ,\phi )+\Omega (\theta )\right]}^{2}\right\},$$

where $\Lambda^{\left(1\right)}\left(\theta ,\phi \right)$ and $\Omega^{\left(1\right)}\left(\theta \right)$ represents the first derivatives of Λ(θ,ϕ) and Ω(θ), respectively. These can be easily obtained by standard procedures, which after being evaluated at θ=0 are reduced to:

(21) $$\Lambda^{\left(1\right)}\left(0\right)=\sum_{n=0}^{N-1}\left\{\frac{2\sigma_{d}^{2}{\left|x\left[n\right]\right|}^{2}}{\sigma^{4}}-\frac{{2|t\left[n-\tau \right]|}^{2}}{\sigma^{2}}\right\},$$

and

(22) $$\Omega^{\left(1\right)}\left(0\right)=-\frac{2N\sigma_{d}^{2}}{\sigma^{2}}.$$

Therefore, $p^{\left(2\right)}\left(x;\theta ,\phi \right)$ evaluated at θ=0 is given by

(23) $$\begin{array}{ccc} {\left.p^{\left(2\right)}\left(x;\theta ,\phi \right)\right|}_{\theta =0} & = & p\left(x;0\right)\left[\sum_{n=0}^{N-1}\left\{\frac{2\sigma_{d}^{2}{\left|x\left[n\right]\right|}^{2}}{\sigma^{4}}-\frac{{2|t\left[n-\tau \right]|}^{2}}{\sigma^{2}}\right\}\right.\\ & & \left.-\frac{2N\sigma_{d}^{2}}{\sigma^{2}}+{\left(\frac{2}{\sigma^{2}}\sum_{n=0}^{N-1}ℜ\left\{e^{j\phi }t\left[n-\tau \right]x^{*}\left[n\right]\right\}\right)}^{2}\right]. \end{array}$$

Next, by substituting (23) in (14) and then including the resulting constant terms along with $\lambda_{st}$ in a new threshold $\lambda_{st}^{\prime }$, the second derivative-based version of the ST-Det (or simply ST-Det) that depends on the unknown values ϕ and τ is:

(24) $$\Phi_{{\mathrm{ST}}_{2}}\left(x,\phi ,\tau \right)=\sum_{n=0}^{N-1}{\left|x\left[n\right]\right|}^{2}+\frac{2}{\sigma_{d}^{2}}{\left(\sum_{n=0}^{N-1}ℜ\left\{e^{j\phi }t\left[n-\tau \right]x^{*}\left[n\right]\right\}\right)}^{2}>\lambda_{st}^{\prime }.$$

**Figure 4:**
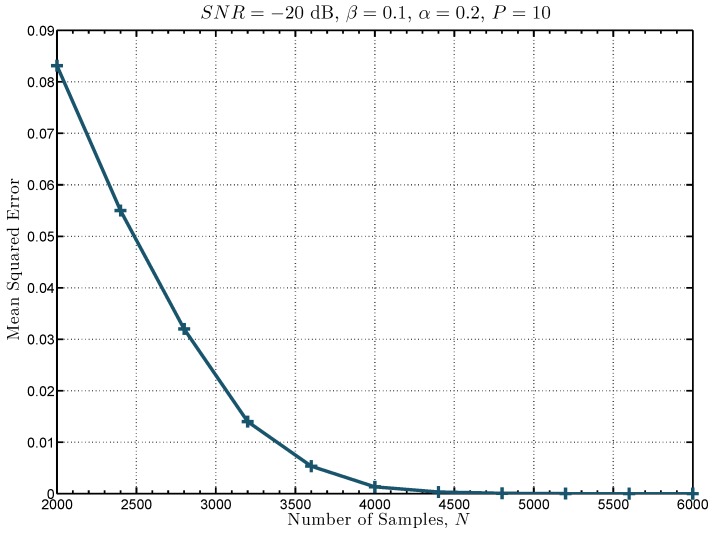
Mean squared error of the power function of the ST-based detector (ST-Det) test statistic against the number of samples *N*.

After substituting the estimate of τ modulo-P, which is obtained solving (7), and then, the estimate of ϕ given by (19), the enhanced ST-Det test statistic is finally given by

(25) $$\Phi_{\mathrm{ST}}\left(x\right)=\underset{\Phi_{\mathrm{ed}}}{\underset{︸}{\sum_{n=0}^{N-1}{\left|x\left[n\right]\right|}^{2}}}+\frac{2}{\sigma_{d}^{2}}\underset{\Phi_{t}}{{\left|\underset{︸}{\sum_{n=0}^{N-1}t\left[n-\hat{\tau }\right]x^{*}\left[n\right]}\right|}^{2}}>\lambda_{st}^{\prime },$$

where $\Phi_{\mathrm{ed}}$ denotes the energy detection metric of the ST-Det and $|\Phi_{t}{|}^{2}$ denotes the ST related metric of the ST-Det. It is important to mention that the optimality of the detector in (25) might have been lost due to the estimates $\hat{\tau }$ and $\hat{\phi }$. In order to evaluate the difference between the test statistic based on the exact values of τ and ϕ (i.e., $\Phi_{{\mathrm{ST}}_{2}}\left(x,\phi ,\tau \right)$) and the test statistic based on the estimated values $\hat{\tau }$ and $\hat{\phi }$ (i.e., $\Phi_{\mathrm{ST}}\left(x\right)$), the power of each test defined respectively as $\mathrm{Pr}\{\Phi_{{\mathrm{ST}}_{2}}\left(x,\phi ,\tau \right)>\lambda_{st}^{\prime }|H_{1}\}$ and $\mathrm{Pr}\{\Phi_{\mathrm{ST}}\left(x\right)>\lambda_{st}^{\prime }|H_{1}\}$, can be considered. For this, 5000 Monte Carlo simulations are performed to quantify the mean squared error between the power of the test statistic in (24) and the power of the test statistic in (25) as shown in Figure 4. It can be seen in this figure that the mean squared error is very close to zero when the number of samples *N* used for spectrum sensing is greater than 4000. For example, the mean squared error is $2.53\times 10^{-6}$ when N=6000, which is the number of samples required to attain a low synchronization error as shown in Figure 3. Therefore, since simulations show that the difference between the power of both test statistics is very small, the proposed detector in (25) continues to be referred to as locally optimum.

Thus, in the second sample processing period, an enhanced version of ST-training based detection is used, since both the training and data sequences are exploited. Interestingly, in the very low SNR region, the ST related term of the ST-Det plays a major role in the detection of ST signals, since $\sigma_{d}^{2}$ is very small with respect to $\sigma^{2}$. Moreover, the ST-Det can also be used to detect non-ST signals, given that it reduces to the energy detection metric when t[n−τ]=0∀n. Additionally, the algorithm is relatively simple and its implementation in an SU terminal is feasible. Although higher order derivatives of p(x,θ,ϕ) can be used in (14) to obtained the test statistic of the ST-Det, in this paper, the detector in (25) is used to analyze the performance of the proposed SCAR-Phase of spectrum sensing. The detection performance metrics of the ST-Det are presented below.

#### 3.2.1. ST-Det Probability of a False Alarm

The probability of a false alarm of the ST-Det, denoted by $P_{\mathrm{fa}}^{st}$ is defined as the probability of deciding $H_{1}$ when $H_{0}$ is true, i.e.,

(26) $$P_{\mathrm{fa}}^{st}≜\mathrm{Pr}\left\{\Phi_{\mathrm{ST}}\left(x\right)>\lambda_{st}^{\prime }|H_{0}\right\}=\int_{\lambda^{\prime }}^{\infty }p_{\Phi_{\mathrm{ST}}}\left(x|H_{0}\right)dx,$$

where $p_{\Phi_{\mathrm{ST}}}\left(x|H_{0}\right)$ is the PDF of $\Phi_{\mathrm{ST}}\left(x\right)$ when $H_{0}$ is true. In order to obtain an analytical expression for the $P_{\mathrm{fa}}^{st}$, a statistical analysis must be carried out on $\Phi_{\mathrm{ST}}\left(x\right)$ when $H_{0}$ is true. Note that, for a large *N*, the central limit theorem (CLT) can be invoked. Hence, $\Phi_{\mathrm{ed}}$ is Gaussian distributed under $H_{0}$. Therefore, after analyzing its statistical properties it can be determined that

(27) $$\Phi_{\mathrm{ed}}∼N\left(N\sigma^{2},N\sigma^{4}\right).$$

Similarly, $\Phi_{t}$ is a complex Gaussian random variable (r.v.) Then, after computing its mean and variance it can be shown that

(28) $$\Phi_{t}∼\mathrm{CN}\left(0,N\sigma^{2}\sigma_{t}^{2}\right).$$

Thereby, $|\Phi_{t}{|}^{2}$ can be expressed in terms of a chi-squared distribution with two degrees of freedom (i.e., exponential distribution). Therefore, under $H_{0}$, the ST-Det test statistic can also be written as:

(29) $$\Phi_{\mathrm{ST}}\left(x\right)=\psi G+N\sigma^{2}+\upsilon E,$$

where G∼N(0,1), $\psi =\sigma^{2}\sqrt{N}$, E is an exponential r.v with parameter ρ=1/2 (i.e. E∼Exp(1/2)) and $\upsilon =N\sigma^{2}\alpha$. In strict sense, G and E are not independent, which complicates obtaining the exact distribution of $\Phi_{\mathrm{ST}}\left(x\right)$. However, since it can be shown that correlation amongst these random variables is low, independence can be assumed without affecting the results significantly. Considering this, the expression for $P_{\mathrm{FA}}$ is found as:

(30) $$P_{\mathrm{fa}}^{st}=\int_{0}^{\infty }\int_{\lambda_{st}^{\prime }}^{\infty }\frac{1}{\psi \sqrt{2\pi }}e^{-\frac{1}{2}{\left(\frac{G-N\sigma^{2}-\upsilon E}{\psi }\right)}^{2}}\frac{1}{2}e^{-E/2}dGdE.$$

Solving the integral, the expression for the probability of false alarm is:

(31) $$P_{\mathrm{fa}}^{st}=f\left(z\right):=\exp\left(\frac{\psi^{2}-4\upsilon z}{8\upsilon^{2}}\right)Q\left(\frac{\psi^{2}-2\upsilon z}{2\upsilon \psi }\right)+Q\left(\frac{z}{\psi }\right),$$

where $z=\lambda_{st}^{\prime }-N\sigma^{2}$. Considering that f(z) is monotonically decreasing in *z*, the threshold can be obtained as

(32) $$\lambda_{st}^{\prime }=f^{-1}\left(P_{\mathrm{fa}}^{st}\right)+N\sigma^{2},$$

where $f^{-1}\left(\cdot \right)$ is the inverse function of f(·).

#### 3.2.2. ST-Det Probability of Detection

The probability of detection of the ST-Det, denoted by $P_{d}^{st}$ is defined as the probability of deciding $H_{1}$ when $H_{1}$ is true, i.e.,

(33) $$P_{d}^{st}≜\mathrm{Pr}\left\{\Phi_{\mathrm{ST}}\left(x\right)>\lambda_{st}^{\prime }|H_{1}\right\}=\int_{\lambda_{st}^{\prime }}^{\infty }p_{\Phi_{\mathrm{ST}}}\left(x|H_{1}\right)dx,$$

where $p_{\Phi_{\mathrm{ST}}}\left(x|H_{1}\right)$ is the PDF of the test statistic $\Phi_{\mathrm{ST}}\left(x\right)$ when $H_{1}$ is true. Next, $P_{d}^{st}$ is obtained by analyzing the statistical properties of $\Phi_{\mathrm{ST}}\left(x\right)$ under $H_{1}$. Using the CLT, it can be shown that

(34) $$\Phi_{\mathrm{ed}}∼N\left(N\theta^{2}\left(\sigma_{d}^{2}+\sigma_{t}^{2}\right)+N\sigma^{2},\;2N\theta^{2}\sigma_{t}^{2}\left(\theta^{2}\sigma_{d}^{2}+\sigma^{2}\right)+N{\left(\theta^{2}\sigma_{d}^{2}+\sigma^{2}\right)}^{2}\right)$$

and

(35) $$\Phi_{t}∼\mathrm{CN}\left(N\theta e^{-j\phi }\sigma_{t}^{2},\;N\sigma_{t}^{2}\left(\theta^{2}\sigma_{d}^{2}+\sigma^{2}\right)\right),$$

when $\hat{\tau }=\tau$ modulo-P. Hence, $|\Phi_{t}{|}^{2}$ follows a non central chi squared distribution with two degrees of freedom and a non centrality parameter given by $\rho =2N\theta^{2}\sigma_{t}^{2}/\left(\theta^{2}\sigma_{d}^{2}+\sigma^{2}\right)$. Therefore, under hypothesis $H_{1}$, the ST-Det test statistic can be written as:

(36) $$\Phi_{\mathrm{ST}}\left(x\right)=\psi_{{}_{1}}G+N\theta^{2}\left(\sigma_{d}^{2}+\sigma_{t}^{2}\right)+N\sigma^{2}+\upsilon_{{}_{1}}X,$$

where $\psi_{{}_{1}}=\sqrt{2N\theta^{2}\sigma_{t}^{2}\left(\theta^{2}\sigma_{d}^{2}+\sigma^{2}\right)+N{\left(\theta^{2}\sigma_{d}^{2}+\sigma^{2}\right)}^{2}}$, $\upsilon_{{}_{1}}=N\alpha \left(\theta^{2}\sigma_{d}^{2}+\sigma^{2}\right)$, G∼N(0,1) and X is a non central chi squared r.v with two degrees of freedom and non centrality parameter ρ. By assuming independence amongst these random variables the $P_{d}^{st}$ is found as:

(37) $$P_{d}^{st}=\int_{0}^{\infty }\int_{\lambda_{st}^{\prime }}^{\infty }\frac{1}{\psi_{{}_{1}}\sqrt{2\pi }}e^{-\frac{1}{2}{\left(\frac{G-\varsigma }{\psi_{{}_{1}}}\right)}^{2}}\frac{1}{2}e^{-\frac{1}{2}\left(X+\rho \right)}I_{0}\left(\sqrt{\rho X}\right)dGdX,$$

where $\varsigma =N\theta^{2}\left(\sigma_{d}^{2}+\sigma_{t}^{2}\right)+N\sigma^{2}+\upsilon_{{}_{1}}X$ and $I_{0}\left(\cdot \right)$ is the modified Bessel function of the first kind and order 0. Since finding a closed-form solution of (37) makes the analysis more involved, the $P_{d}^{st}$ is found by noting that the inner integral is the Q-function and then evaluating the resulting outer integral by relying on numerical methods, e.g., the trapezoidal method over a large number of evenly spaced points.

## 4. Performance Metrics for the SCAR-Phase of Spectrum Sensing

### 4.1. Detection Performance

The detection performance of the SCAR-Phase of Spectrum Sensing is characterized in terms of the overall probability of false alarm and the overall probability of detection. Since this approach combines two detectors as in [26], a false alarm occurs in the following two cases:

(i) $\Phi_{ed}\left(x\right)>\lambda_{ed}$ when $H_{0}$ is true.
(ii) $\Phi_{\mathrm{ST}}\left(x\right)>\lambda_{st}^{\prime }$ given that $\Phi_{ed}\left(x\right)\leq \lambda_{ed}$ when $H_{0}$ is true.

Therefore, the overall probability of false alarm, denoted by $P_{\mathrm{FA}}$, of the SCAR-Phase of spectrum sensing is:

(38) $$P_{\mathrm{FA}}=P_{\mathrm{fa}}^{ed}+\left(1-P_{\mathrm{fa}}^{ed}\right)P_{\mathrm{fa}}^{st}.$$

Whereas a correct detection occurs in the following two cases:

(i) $\Phi_{ed}\left(x\right)>\lambda_{ed}$ when $H_{1}$ is true.
(ii) $\Phi_{\mathrm{ST}}\left(x\right)>\lambda_{st}^{\prime }$ given that $\Phi_{ed}\left(x\right)\leq \lambda_{ed}$ when $H_{1}$ is true.

Hence, the overall probability of detection of the SCAR-Phase of spectrum sensing, denoted by $P_{D}$, is:

(39) $$P_{D}=P_{d}^{ed}+\left(1-P_{d}^{ed}\right)P_{d}^{st}.$$

Note that both $P_{\mathrm{FA}}$ and $P_{D}$ depend on the values $\lambda_{ed}$ and $\lambda_{st}$. Consequently, the values of both thresholds that maximizes $P_{D}$ subject to a false alarm rate constraint β must be determined. Since the optimal $P_{D}$ is attained by $P_{\mathrm{FA}}=\beta$, as demonstrated in [26], this problem can be written as:

(40) $$\begin{array}{c} \underset{\left(\lambda_{ed},\;\lambda_{st}^{\prime }\right)}{\max\;}P_{D}\left(\lambda_{ed},\lambda_{st}^{\prime }\right)\\ s.t.\;P_{\mathrm{FA}}=\beta . \end{array}$$

From (38) and a given false alarm rate constraint β, the probability of false alarm for the energy detector is $P_{\mathrm{fa}}^{ed}=\left(\beta -P_{\mathrm{fa}}^{st}\right)/\left(1-P_{\mathrm{fa}}^{st}\right)$. Hence, using (10), $\lambda_{ed}$ can be written as a function $g(\lambda_{st}^{\prime })$, i.e.,

(41) $$\lambda_{ed}=g\left(\lambda_{st}^{\prime }\right)=\sqrt{N\sigma^{4}}Q^{-1}\left(\frac{\beta -P_{\mathrm{fa}}^{st}}{1-P_{\mathrm{fa}}^{st}}\right)+N\sigma^{2}.$$

Therefore, as shown in [26], the optimization problem in (40) can be simplified as follows:

(42) $$\underset{\lambda_{st}^{\prime }}{\max\;}P_{D}\left(g\left(\lambda_{st}^{\prime }\right),\lambda_{st}^{\prime }\right).$$

Thus, from (42) and (41) the optimal $\lambda_{st}^{\prime }$ and $\lambda_{ed}=g\left(\lambda_{st}^{\prime }\right)$ can be found.

### 4.2. Mean Computational Complexity

In this section, the computational complexity is measured in terms of the total number of real operations (additions and multiplications) needed to decide the presence of the PU’s signal. As shown in the block diagram displayed in Figure 2, enhanced ST-based detection is only performed if the energy detector does not detect a PU’s signal. Using the energy detector, deciding the absence or presence of the PU’s signal can be modeled as a Bernoulli r.v. denoted by *B*, whose probability of deciding the absence of the PU’s signal (denoted by $P_{H_{0}}$) is given by $P_{H_{0}}=\mathrm{Pr}\left\{H_{0}\right\}\left(1-P_{\mathrm{fa}}^{ed}\right)+\mathrm{Pr}\left\{H_{1}\right\}\left(1-P_{d}^{ed}\right)$, where $\mathrm{Pr}\left\{H_{0}\right\}$ and $\mathrm{Pr}\left\{H_{1}\right\}$ are the prior probabilities denoting the absence and presence of the PU in the frequency band, respectively. Hence, carrying out the second phase of sample processing depends on the value that this r.v takes (i.e., its expected value, given by $E\left[B\right]=P_{H_{0}}$). Hence, the total mean computational complexity of the SCAR-Phase of spectrum sensing is:

(43) $$\bar{C}=C_{sync}+C_{ed}+P_{H_{0}}C_{st},$$

where $C_{sync}$, $C_{ed}$ and $C_{st}$ are the number of real operations required by the synchronization process, the energy detector and the enhanced ST-based detection. Note that $C_{sync}$ and $C_{st}$ are related to the method used to carry out the synchronization process and the enhanced ST-based detection. To obtain the number of real operations, it is considered that in a modern digital signal processor (DSP), the time consumed by a real multiplication and a real addition is the same. Moreover, it is considered that a complex addition requires two real additions and a complex multiplication needs four real multiplications and two real additions. Hence, for the synchronization method previously described, which requires $P^{3}+2P+3$ complex operations [18], $C_{sync}=216P^{3}+12P+18$ real operations. Moreover, the energy detector requires *N* complex multiplications and N−1 real additions. Hence, $C_{ed}=7N-1$ real operations [19]. For the enhanced ST-based detection, the ST-related metric of the ST-Det, $\Phi_{t}$, needs N+1 complex multiplications and N−1 complex additions, i.e., 4N+4 real multiplications and 4N real additions. Therefore, $\Phi_{\mathrm{ST}}\left(x\right)$ requires 8N+5 real multiplications and 7N real additions. Thus, $C_{st}=15N+5$ real operations. Finally, the mean computational complexity of the SCAR-Phase of spectrum sensing is:

(44) $$\bar{C}=216P^{3}+12P+17+7N+P_{H_{0}}\left(15N+5\right).$$

### 4.3. Analysis of a Secondary User’s Throughput

In periodic spectrum sensing, the frame structure for the SU’s network consists of one spectrum sensing phase followed by one transmission phase in which the SU transmits data if the PU is inactive. Considering that the total number of samples of a frame is $N_{f}$, the average throughput for the SU’s network is given by [27]

(45) $$R\left(N\right)=\left(\frac{N_{f}-N}{N_{f}}\right)C_{0}\left(1-P_{\mathrm{FA}}\right)\mathrm{Pr}\left\{H_{0}\right\}+\left(\frac{N_{f}-N}{N_{f}}\right)C_{1}\left(1-P_{D}\right)\mathrm{Pr}\left\{H_{1}\right\},$$

where $N_{f}-N$ is the length of the data transmission phase. Moreover, $C_{0}=\log_{2}\left(1+\gamma_{s}\right)$ is the throughput of the SU’s network when the SU transmits under $H_{0}$ with $\gamma_{s}$ denoting the SNR of the secondary link, whereas $C_{1}=\log_{2}\left(1+\frac{\gamma_{s}}{1+\gamma_{p}}\right)$ is the throughput of the SU’s network when the SU transmits under $H_{1}$ with $\gamma_{p}$ denoting the SNR of the link between the PU and the SU. Note that *R* depends on the value of *N*, hence, the optimal value of *N* that maximizes the achievable SU’s throughput for a given target probability of detection, $\beta_{d}$, must be found. In this paper, it is used (45) to obtain the SU’s throughput for a determined value of $\beta_{d}$.

## 5. Simplified SCAR-Phase of Spectrum Sensing

**Figure 5:**
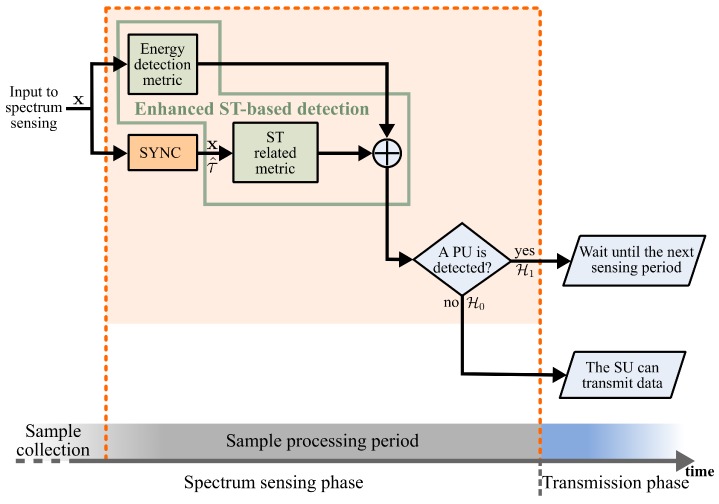
Block diagram of the simplified SCAR-Phase spectrum sensing.

The simplified SCAR-Phase of spectrum sensing block diagram is shown in Figure 5. Different to the SCAR-Phase sensing, the simplified version consists of a sample collection period and only one sample processing period. This is due to the fact that the contribution of the energy detection metric in the enhanced ST-based detection (ST-Det) in (25) does not require the synchronization procedure. Moreover, the results of this metric are the same as in the first sample processing period of the SCAR-Phase sensing, thus this period can be omitted. Hence, after the SU collects the samples, the synchronization process with the transmitted PU’s sequence is carried out at the same time that the energy of the received signal is calculated. Then, once τ is estimated, the already synchronized ST sequence can be used in the ST-related metric of the ST-Det. If the enhanced ST-based detector decides that the PU’s signal is present, the SU waits until the next spectrum sensing phase, or else, the SU can transmit data. Note that simplifying the sensing phase to one sample processing period reduces the computational complexity of the approach, which is characterized in terms of the total number of real operations needed to make a decision.

After analyzing the mathematical structure of the proposed simplified SCAR-Phase of spectrum sensing, the computational complexity is found to be $C=C_{sync}+C_{ed}+C_{t}$, where the number of real operations needed by the synchronization method is $C_{sync}=216P^{3}+12P+18$, by the energy detection metric is $C_{ed}=7N-1$. Therefore,

(46) $$C=216P^{3}+12P+15N+23.$$

Next, the detection performance metrics of the simplified SCAR-Phase sensing are presented. The overall probability of false alarm is:

(47) $$P_{\mathrm{FA}}=P_{\mathrm{fa}}^{st},$$

where $P_{\mathrm{fa}}^{st}$ is given by (31).

Additionally, the overall probability of detection, when $\hat{\tau }=\tau$ modulo-P, is:

(48) $$P_{D}=P_{d}^{st},$$

where $P_{d}^{st}$ is given by (37).

**Figure 6:**
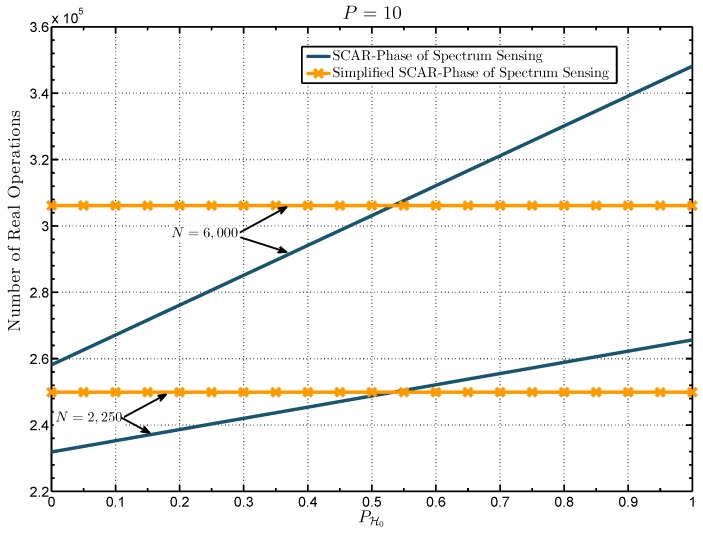
Computational complexity in terms of number of real operations against $P_{H_{0}}$ for a fixed value of number of samples *N*.

In Table 1 the computational complexity of the proposed approaches and the energy detector can be compared. Note that, for the same number of samples, the computational complexity of the energy detector is the lowest. Although the computational complexity of the energy detector is much simpler in contrast to the proposed approaches, the detection performance of the SCAR-Phase sensing and its simplified version is much higher in the very low SNR region, as it will be shown later in the results section, which makes it worth the increase in computational complexity. Additionally, since the number of real operations used by the SCAR-Phase of spectrum sensing depends on the value of $P_{H_{0}}$, its computational complexity will be greater than that of the simplified scheme as $P_{H_{0}}$ approaches 1. This is shown in Figure 6, where the number of real operations of the proposed schemes as a function of $P_{H_{0}}$ is shown for a fixed value of *N*. Note in this figure that the computational complexity of the simplified SCAR-Phase of spectrum sensing remains constant, whereas for the SCAR-Phase of spectrum sensing increases according to $P_{H_{0}}$. It can be seen that for $P_{H_{0}}$ values greater than ≈0.55 the computational complexity of the proposed simplified approach is lower than that of the SCAR-Phase of spectrum sensing. This makes sense given that the probability of executing the second processing period of the SCAR-Phase sensing is greater. In the next section, it will be shown that to attain a given detection performance in the very low SNR region, the computational complexity of the simplified version is lower than that of the SCAR-Phase of spectrum sensing.

## 6. Results and Discussion

In this section, the performance of the proposed SCAR-Phase of spectrum sensing and its simplified version are analyzed via Monte Carlo simulations. The number of Monte Carlo iterations is set to 5000. In the considered CR scenario, the PU transmits an ST-based signal given by (1). The ST sequence is designed using (8) with a TIR value α=0.2, a training period P=10 and considering that $\sigma_{d}^{2}+\sigma_{t}^{2}=1$. The received signal at the SU is modeled as in (2), with random channel gains and τ selected from the interval [0,P−1] in such a way that the time offset variate in each Monte Carlo simulation. Hence, $\hat{\tau }$ is obtained in each trial using (7), thus the Monte Carlo simulations are carried out considering the corresponding synchronization errors for each SNR value. The noise variance is chosen to satisfy a determined value of instantaneous SNR (denoted by γ), hence $\sigma^{2}=\theta^{2}\left(\sigma_{d}^{2}+\sigma_{t}^{2}\right)/\gamma$.

The region of interest is the low SNR between the PU and the SU, where the detection of the PU’s signal can be tough. Given that, in such a scenario, the number of samples required by the energy detector dramatically increases at the time that the SNR decreases, the SUs’ throughput is severely affected. Therefore, both proposed approaches are compared with the traditional energy detector, since their aim is to reduce the number of samples used in the spectrum sensing phase to increase the maximum SUs’ throughput when operating in the low SNR region. In order to carry out the Monte Carlo simulations, the values of $\lambda_{st}^{\prime }$ and $\lambda_{ed}=g\left(\lambda_{st}^{\prime }\right)$ that maximize the overall probability of detection ($P_{D}$) for a target $P_{\mathrm{FA}}=\beta$ must be determined for the SCAR-Phase of spectrum sensing. For this purpose, $P_{D}$ is first obtained as a function of $\lambda_{st}^{\prime }$ using (39) and (41). Then, the value $\lambda_{st}^{\prime }$ that maximizes $P_{D}$ is found and used to obtain $\lambda_{ed}=g\left(\lambda_{st}^{\prime }\right)$.

**Figure 7:**
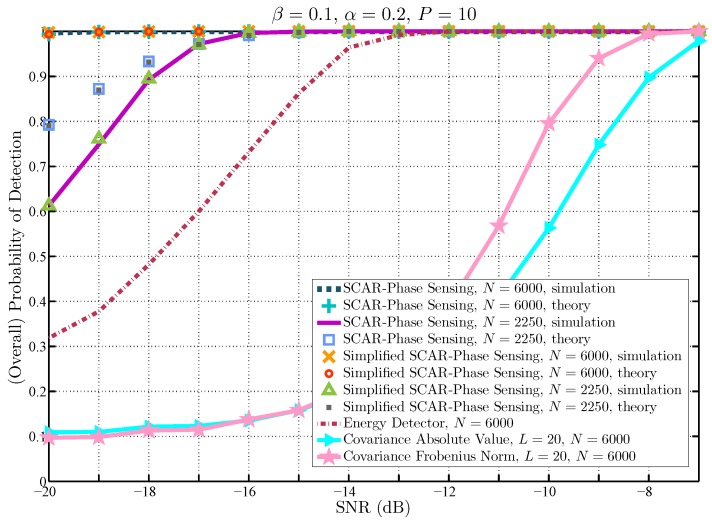
Comparison of the (overall) probability of detection against SNR for different spectrum sensing schemes.

The detection performance of the proposed SCAR-Phase and simplified SCAR-Phase of spectrum sensing is shown in Figure 7, in terms of the attained overall probability of detection (satisfying a constraint on the overall probability of false alarm β=0.1) as a function of SNR. These results are obtained by finding the number of samples used for spectrum sensing, *N*, that attains an objective probability of synchronization error, below which the theoretical expression of the overall probability of detection is accurate enough. For example, with N=6000 the probability of synchronization error is $2\times 10^{-2}$ at −20 dB (see Figure 3 for reference). Therefore, for a set of collected samples of length N≥6000, the probability of synchronization error will be less than $2\times 10^{-2}$ in the low SNR region above −20 dB. Hence, the theoretical results for the SCAR-Phase of spectrum sensing and the simplified version approximate the simulation results, as shown in Figure 7. Moreover, in order to provide a fair perspective on the performance of the proposed approaches, it is also shown in this figure the attained overall probability of detection against SNR with a number of samples that produce a higher probability of synchronization error. For example, with N=2250 the probability of synchronization error is approximately 0.28 at −20 dB (see Figure 3 for reference). Thus, it can be seen in Figure 7 that with N=2250 the theoretical approximations (in both proposed approaches) do not perfectly match the simulation results as the SNR decreases. Indeed, in this case, the theoretical results overestimate the simulations in the very low SNR since more synchronization errors are made. It is worth mentioning that the results of both proposed approaches are similar, given the fact that in the low SNR the SCAR-Phase sensing results rely mostly on the ST-Det of the second sample processing period, whereas the simplified SCAR-Phase sensing results rely only on the ST-Det in the unique sample processing period. Additionally, for comparison purposes, Figure 7 also exhibits the theoretical detection performance of the energy detector with N=6000, which is the number of samples used by the proposed approaches. For the results labeled as energy detector, the theoretical expressions in (10)–(12) are used. It can be noticed that the performance of the SCAR-Phase of spectrum sensing and the simplified version evince a similar operation in comparison to the energy detector in the SNR region above −13 dB. However, for SNR values below −13 dB, the second sample processing period of the SCAR-Phase sensing is enabled to exploit the ST sequence by means of the ST-Det. Therefore, in the low SNR, the performance of the SCAR-Phase sensing is significantly improved with respect to that of the energy detector. In consequence, for a target detection performance, the number of samples can be reduced in the low SNR when the proposed approaches are implemented. Finally, note in this figure that spectrum algorithms that are based on the sample covariance matrix, such as the Covariance Absolute Value and Covariance Frobenius Norm [7], have been included for comparison purposes under the signal model considered in (2). It can be seen that they exhibit a degradation in detecting uncorrelated signals and their performance drastically deteriorates since *N* is small [7], which is the scenario analyzed in this paper. Therefore, further analyses compare the proposed approaches only with the energy detector.

**Figure 8:**
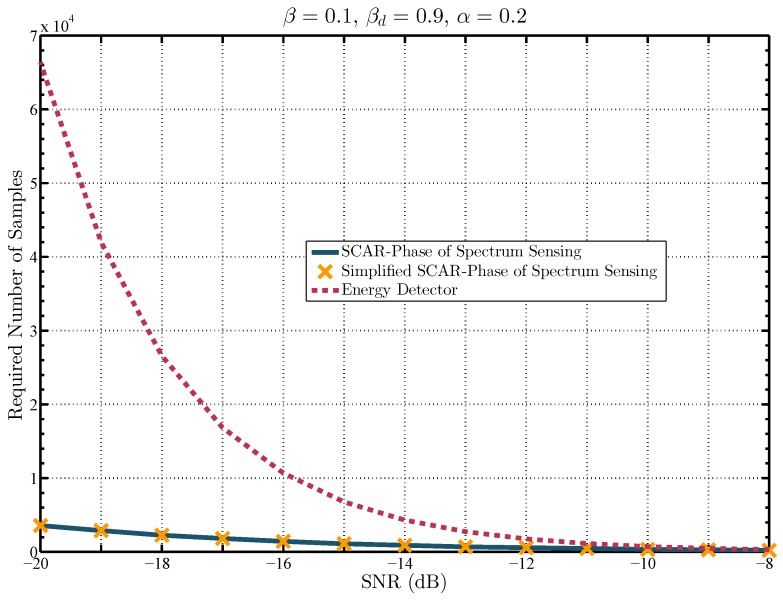
Comparison of the required number of samples to achieve a probability of detection $\rho_{d}=0.9$ for a target probability of false alarm ρ=0.1 for different spectrum sensing schemes.

Next, Figure 8 shows the required number of samples as a function of the SNR, for a given pair of target probabilities of detection and false alarm, $\beta_{d}=0.9$ and β=0.1, respectively. It quantifies the increasing difference in the required number of samples to achieve the target probabilities as the SNR decreases. For example, for an SNR of −16 dB, the SCAR-Phase of spectrum sensing and the simplified SCAR-Phase of spectrum sensing need approximately $2\times 10^{3}$ samples whilst the energy detector requires approximately $1\times 10^{4}$ samples, which is around five times the number of samples required by the proposed approaches. Furthermore, when the SNR is equal to −20 dB, the new schemes only require approximately $4\times 10^{3}$, whereas the energy detector requires approximately $6.6\times 10^{4}$ samples, which is 16.5 times the required number of samples by the proposed approaches. Moreover, Figure 8 evidences that as the SNR increases, the detection performance of the energy detector and the proposed approaches is similar. Note that, the SCAR-Phase of spectrum sensing and the simplified SCAR-Phase of spectrum sensing requires the same number of samples. However, the simplified version of the SCAR-Phase is in the sense of computational complexity, which is analyzed in terms of the number of real operations in what follows.

**Figure 9:**
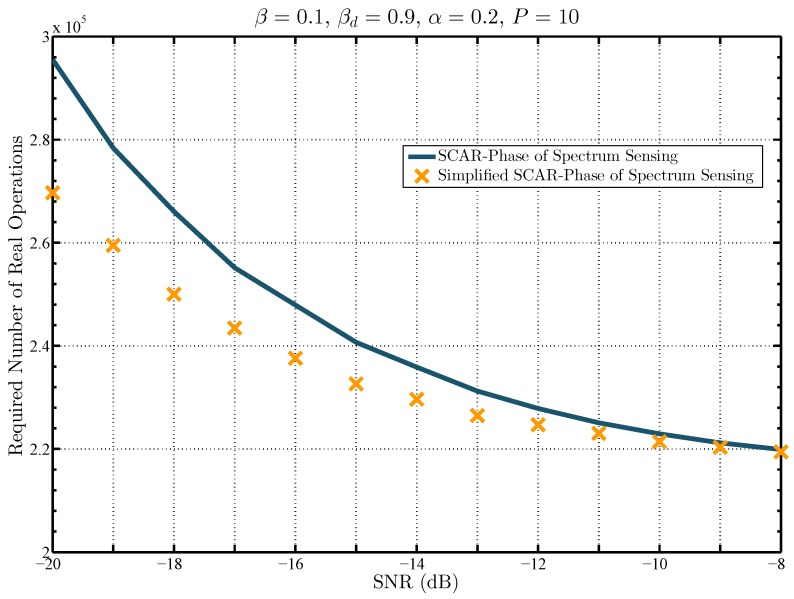
Comparison of the required number of real operations to achieve a probability of detection $\beta_{d}=0.9$ for a target probability of false alarm β=0.1 for the proposed spectrum sensing schemes.

The computational complexity of the proposed approaches is shown in Figure 9 in terms of the required number of real operations as a function of the SNR. The results are obtained for a target pair of probabilities $\beta_{d}=0.9$ and β=0.1 using (44) for the SCAR-Phase sensing and (46) for the simplified version. It can be seen that, as the SNR decreases, the required number of real operations of the simplified SCAR-Phase also decreases in comparison to those required by the other approach. This is due to the fact that in the low SNR, the energy detector in the first sample processing period of the SCAR-Phase sensing decides more frequently the absence of the PU’s signal. In consequence, the enhanced ST-based detection in the second sample processing period is executed. Since in the simplified version, the first sample processing period is omitted, the required number of real operations is reduced. For example, in SNR equal to −15 dB, the SCAR-Phase sensing requires $2.551\times 10^{5}$ real operations whereas the simplified version needs $2.434\times 10^{5}$ real operations. The reduction is more noticeable in lower SNR values. For example, in SNR equal to −20 dB, the SCAR-Phase sensing requires $2.955\times 10^{5}$ real operations whereas the simplified version needs $2.697\times 10^{5}$ real operations.

**Figure 10:**
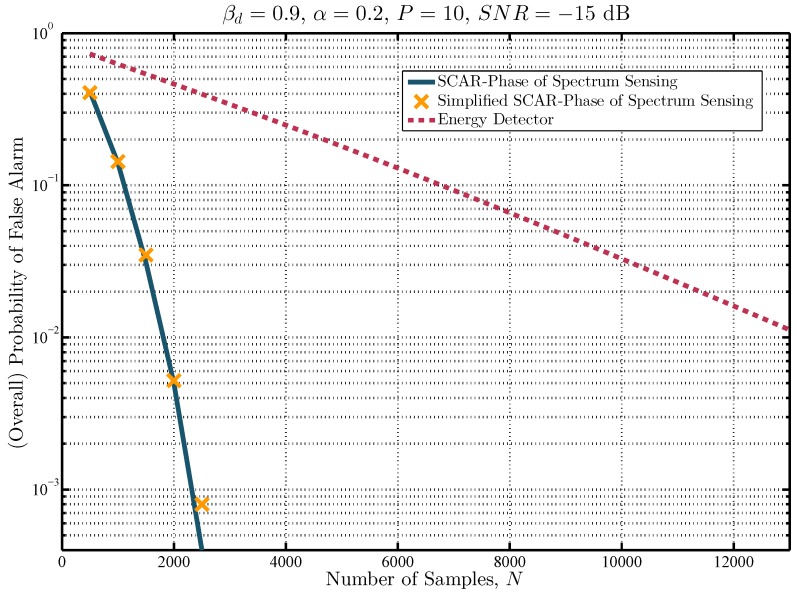
Probability of false alarm against sensing time.

Furthermore, in Figure 10 the performance of the proposed approaches is compared to the energy detector with a constraint on the level of protection for the PU. Therefore, the overall probability of a false alarm is obtained against different number of samples to satisfy a $\beta_{d}=0.9$. The results show that with N=2000, the probability of false alarm for the proposed approaches is approximately 0.0052, whilst for the energy detector, it is equal to 0.4633. This difference increases with the number of samples used for spectrum sensing, impacting the SU’s achievable throughput.

**Figure 11:**
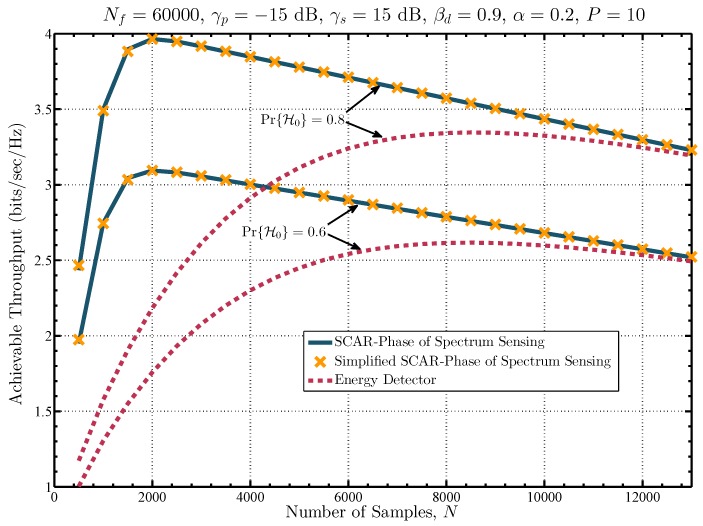
Achievable throughput for the secondary network.

Finally, the maximum SU’s achievable throughput against the number of samples is compared in Figure 11 for the proposed methods and the energy detector. The results are obtained using (45) for a frame size of $N_{f}=60,000$ samples, $\gamma_{s}=15$ dB and $\gamma_{p}=-15$ dB. Moreover, different values of $\mathrm{Pr}\{H_{0}\}$ are considered to show the dependency of the SU’s achievable throughput on the value of the prior probability of absence of the PU. It can be seen that for the proposed approaches the maximum throughput is achieved with *N* = 2000 samples, whereas for the energy detector it is achieved with N≈8500 samples. Note that these number of samples are equal for both prior probabilities, but the achievable throughput varies accordingly since there are more opportunities to carry out SU’s transmissions when the probability that the PU is inactive is greater.

## 7. Conclusions

A new approach for the exploitation of ST PUs’ systems in the context of spectrum sensing for CR was presented. The proposed SCAR-Phase sensing took advantage of the ST information to carry out the synchronization of an SU with the ST PU’s sequence and also to enhance its detection performance. This was possible by splitting the spectrum sensing phase into one sample collection and two sample processing periods. In the first one, the synchronization with the ST sequence was achieved by means of a robust algorithm with a small probability of error in the low SNR region. Then, in the second one, an enhanced ST-based detector (i.e., ST-Det) was designed to perform the spectrum sensing with a small number of samples and a high probability of detection. This approach is different from the two-stage spectrum sensing scheme, which consists of a short first sample collection plus a large second sample collection with its processing period each. Furthermore, a simplified version of the SCAR-Phase sensing with a reduced computational complexity was introduced by exploiting the high detection performance of the proposed ST-Det. The results showed that, in the low SNR, the proposed approaches exhibit a significantly higher overall probability of detection than the energy detector. Moreover, it was shown that the required number of samples to achieve a target probability of detection is significantly lower for the proposed schemes. Furthermore, the maximum SU’s achievable throughput was quantified and showed to be higher for the SCAR-Phase sensing and the simplified version than that for the energy detector. Further studies need to be carried out in order to assess the performance of the proposed methods in the presence of noise power uncertainty.

## Figures and Tables

**Table 1:** Computational complexity comparison of the SCAR-Phase of spectrum sensing, the simplified SCAR-Phase of spectrum sensing and the energy detector in terms of the number of real operations.

Spectrum Sensing Method	Number of Real Operations
Energy detector	7N−1 [19]
SCAR-Phase of spectrum sensing	$216P^{3}+12P+17+7N+P_{H_{0}}\left(15N+5\right)$
Simplified SCAR-Phase of spectrum sensing	$216P^{3}+12P+15N+23$

## Abbreviations

The following abbreviations are used in this manuscript:

CLT	Central limit theorem
CR	Cognitive radio
DSP	Digital signal processor
LOD	Locally optimum detection
PDF	Probability density function
PU	Primary user
RF	Radio frequency
r.v.	Random variable
SCAR-Phase	Superimposed training combined approach for a reduced phase
SNR	Signal-to-noise ratio
ST	Superimposed training
ST-Det	Superimposed training-based detector
SU	Secondary user
SYNC	Synchronization
TDMT	Time domain multiplexed training
TIR	Training-to-information ratio
